# Melatonin Alleviates Antimony Toxicity by Regulating the Antioxidant Response and Reducing Antimony Accumulation in *Oryza sativa* L.

**DOI:** 10.3390/antiox12111917

**Published:** 2023-10-26

**Authors:** Yutan Chu, Qiongli Bao, Yan Li, Hongyu Sun, Zewei Liu, Jiahao Shi, Yizong Huang

**Affiliations:** 1 Key Laboratory of Original Agro-Environmental Pollution Prevention and Control, Agro-Environmental Protection Institute, Ministry of Agriculture and Rural Affairs, Tianjing 300191, China; 82101202134@caas.cn (Y.C.); 82101191169@caas.cn (Y.L.); 82101201138@caas.cn (H.S.); 82101211166@caas.cn (Z.L.); 82101215316@caas.cn (J.S.); 2 Tianjin Key Laboratory of Agro-Environment and Agro-Product Safety, Agro-Environmental Protection Institute, Ministry of Agriculture and Rural Affairs, Tianjin 300191, China; 3 School of Energy and Environment Science, Yunnan Normal University, Kunming 650500, China

**Keywords:** antimony (Sb), melatonin (MT), rice, antioxidant, AsA–GSH cycle

## Abstract

Antimony (Sb) is a hazardous metal element that is potentially toxic and carcinogenic. Melatonin (MT) is an indole compound with antioxidant properties that plays an essential role in plant growth and alleviates heavy metal stresses. Nevertheless, little is known about the effects and mechanisms of exogenous MT action on rice under Sb stress. The aim of this experiment was to explore the mechanism of MT reducing Sb toxicity in rice via hydroponics. The results showed that Sb stress significantly inhibited the growth of rice, including biomass, root parameters, and root viability. Exogenous MT obviously alleviated the inhibition of Sb stress on seedling growth and increased biomass, root parameters, and root viability by 15–55%. MT significantly reduced the total Sb content in rice and the subcellular Sb contents in roots by nearly 20–40% and 12.3–54.2% under Sb stress, respectively. MT significantly decreased the contents of malondialdehyde (MDA, by nearly 50%), ROS (H_2_O_2_ and O_2_·^−^, by nearly 20–30%), and RNS (NO and ONOO^−^) in roots under Sb stress, thus reducing oxidative stress and cell membrane damage. Furthermore, MT reversed Sb-induced phytotoxicity by increasing the activities of antioxidant enzymes (SOD, POD, CAT, and APX) by nearly 15% to 50% and by regulating the AsA–GSH cycle. In conclusion, this study demonstrates the potential of MT to maintain redox homeostasis and reduce Sb toxicity in rice cells, decreasing the content of Sb in rice and thereby alleviating the inhibition of Sb on rice growth. The results provided a feasible strategy for mitigating Sb toxicity in rice.

## 1. Introduction

Heavy metal pollution is one of the most severe environmental problems facing the world. It causes food security issues and also directly harms animals and human health. As an analogue of arsenic (As), antimony (Sb), a metallic element with potential toxicity and carcinogenicity, is a global pollutant that has been listed as a priority pollutant by many countries [1]. Due to geological processes and anthropogenic activities (such as metal mining, smelting, and coal combustion), Sb soil contamination is becoming increasingly severe, and the concentration of soil Sb in some regions far exceeds the maximum allowable concentration (36 mg kg^−1^) set by the World Health Organization (WHO). For instance, the world’s largest Sb mine is located in China, where the concentration of Sb in the surrounding soil ranges from 10 to 2159 mg kg^−1^ [2]. Excessive Sb in soil causes high Sb concentration in plants and harm to humans and animals [3].

As the most important staple food crop in the world, the average annual production of rice is about 700 million tons [4]. In China, rice cultivation areas account for about 30% of the total food crop areas. Sb enters the human body through rice consumption, ultimately leading to the development of cancer and other diseases [5]. The environmental pollution and ecotoxicity of Sb have attracted a lot of attention in recent years. Sb mainly exists in soil in the form of trivalent Sb (Sb(III)) and pentavalent Sb (Sb(V)); Sb(III) is more easily absorbed by plants than Sb(V) and is more toxic to plants [6]. Many plants can uptake and accumulate Sb in large quantities. The toxic effects of excessive Sb on plants are mainly affect the growth and development of the plants, producing oxidative stress and affecting the absorption of essential nutrients [5].

Plant roots absorb and accumulate most of the Sb, and the cell wall and cytoplasm play an essential part in Sb accumulation in plants [7]. Sb is absorbed and transported by plant tissues via the plastid extracellular or cytoplasmic pathway, where the cell membranes absorb Sb(III) via water channel proteins [8]. It has been shown that Sb stress, especially Sb(III), can interfere with gas exchange and electron transfer in the photosynthetic system of rice and limit photosynthetic efficiency [9]. Excess Sb increases the lipid peroxidation of the cell membranes, produces reactive oxygen species (ROS) and reactive nitrogen species (RNS), and induces oxidative stress [10,11]. In response to the toxicity of antimony, plants have evolved enzymatic and non-enzymatic antioxidant systems to reduce these active substances and thus reduce the absorption or transport of Sb. Sb stress causes a change in cell REDOX (cellular redox) homeostasis, which affects the activities of superoxide dismutase (SOD), peroxidase (POD), catalase (CAT), ascorbate peroxidase (APX), and other enzymes [5]. Glutathione (GSH) and the ascorbic acid (AsA) cycle are the major non-enzymatic antioxidants; the AsA–GSH cycle plays a role in controlling ROS concentrations and resisting heavy metal-induced oxidative stress in plant cells [12]. In the AsA-GSH cycle, APX first uses AsA as the electron donor to catalyze the reduction in H_2_O_2_ to H_2_O, and AsA is oxidized to DHA. Secondly, DHAR with GHS as an electron donor converts DHA back to AsA; GSH disulfide (GSSH) is the oxidized form of GSH, and glutathione reductase (GR) with nicotinamide adenine dinucleotide phosphate (NADPH) as an electron donor converts GSSH back to GSH. Therefore, AsA, DHA, GSH, and GSSH play key roles in protecting plant cells from oxidative damage resulting from heavy metals stress [12].

Plant hormones are small molecules that play a crucial role during all stages of a plant’s life. Melatonin (MT, *N*-acetyl-5-methoxytryptamine), an indole-like natural compound with antioxidant properties, acts as a signaling molecule in the growth and reproduction of plants. MT is an efficient reactive oxygen scavenger and a strong endogenous free radical scavenger. MT regulates plant growth through regulating the antioxidant defense system in plants and interacting with ROS, RNS, and other signaling molecules to participate in plant physiological responses [13], thus mitigating heavy metals stresses. MT promotes plant tolerance to heavy metal stress by improving redox homeostasis, osmotic balance, and primary and secondary metabolism [14]. MT significantly decreases the H_2_O_2_ content, increases the GSH content and the activities of APX and SOD in wheat under Cd stress [15], increases wheat plant growth, and greatly reduces Cd toxicity. Exogenous MT significantly reduced the accumulation of H_2_O_2_ and MDA content and the activities of antioxidant enzymes in rice under Cd and Pb combined stress [16,17]. Soaking seeds with MT could be a potential method to protect wheat seeds from Cr toxicity [18]. Melatonin alleviates nickel toxicity in tomato seedlings by improving photosynthesis and oxidative stress tolerance [19]. In addition, MT participates in the heavy metal transport process in plants and reduces heavy metal transport from plant roots to shoots by improving their morphology and localization in the roots [20], thus alleviating the toxic effects of heavy metals. Melatonin may inhibit the migration of Cd or As ions or regulate the balance of ion and redox state in crops by improving the synthesis of small-molecule chelating agents [21]. However, the role of MT in regulating the growth and stress tolerance of rice under Sb stress remains largely unknown.

In this study, the effects of exogenous MT application on reducing Sb absorption and accumulation in rice seedlings were studied via hydroponics. The indicators regarding rice growth, Sb uptake and transportation, lipid peroxidation, ROS and RNS accumulation, and the antioxidant defense system under MT and/or Sb application were measured to reveal the mitigation mechanism of MT on Sb stress in rice, providing a new strategy for alleviating Sb accumulation in rice.

## 2. Materials and Methods

### 2.1. Plant and Treatment

Grains of Huarun No. 2 were disinfected with 5% hydrogen peroxide (H_2_O_2_) solution for 15 min and then rinsed with deionized water 4 to 5 times. The sterilized seeds were evenly spread on the trays, covered with ultra-pure water, and germinated in a constant temperature incubator at 28 °C. Seeds with a consistent white root appearance were selected for transplanting into black boxes and cultures with 1/10 Hoagland nutrient solution in a chamber. After 2 weeks of cultivation, the seedlings were transferred to black boxes (10.5 cm × 15.5 cm × 17.0 cm, L × W × H). Based on previous experiments (Table S1), 0 and 50 μM Sb concentrations and four MT concentrations (0, 50, 100, and 500 μM) were selected as the treatment concentrations. In total, eight treatments were applied, and four parallels were set for each treatment. Sb was added in the form of C_8_H_4_K_2_O_12_Sb_2_·3H_2_O, and the treatment solution was changed every 2 days. After one week of treatment, the plants were harvested for analysis.

### 2.2. Rice Seedling Growth Measurement

The harvested plants were divided into two parts, and their fresh and dry weight were determined. Their roots were washed three times each with tap water and deionized water. The clear roots were scanned using an automated scanning instrument (Epson Expression 10000XL) and analyzed to determine their root parameters (including length, surface area, volumes, and tip numbers of roots). Root viability was determined using a root viability kit (Shanghai Yuanye Biotechnology Co., Ltd., Shanghai, China). Briefly, 0.5 g fresh samples of the clean roots were incubated in 2,3,5-triphenyl tetrazolium chloride (TTC) solution at a temperature of 37 °C for 1 h. After extraction by grinding with ethyl acetate, the supernatant was collected and then measured according to the manufacturer’s instructions. The root viability index was considered to be the dehydrogenase activity expressed by the amount of reduction in TTC measured at 485 nm.

### 2.3. Analysis of Total Sb Contents in Seedlings

The dried samples were digested at 110 °C for 4 h. A dual-channel atomic fluorescence spectrometer (AFS-9760, Beijing Haiguang Instrument Co., Ltd., Beijing, China) was used to determine the total concentration of Sb at 217.6 nm with the parameters of Sb hollow cathode lamp (HAF-2). The digested liquid’s total Sb concentration was measured using a atomic fluorescence spectrometer (AFS-9760, Beijing, China) with the parameters of the Sb hollow cathode lamp (HAF-2) at 217.6 nm. Each sample was measured three times. Duplicates, method blanks, and Sb certified standard reference material (GBW(E) 100350) from the Centre for Standard Reference of China. were used to ensure quality control during the determination.

### 2.4. Analysis of Sb Subcellular Distributions

The subcellular fractions of the roots were separated into the cell wall, cytosol, and organelles. Briefly, suitable fresh samples were homogenized with an extraction solution (dithiopentetrol, sucrose, and Tris buffer, pH = 7.5) on ice. The homogenate was separated into three fractions using a differential centrifugation technique [22]. The temperature for all extraction processes was 4 °C. Briefly, The homogenate was first centrifuged for 10 min at 3000× *g*, and the precipitated fraction was designated as the cell wall fraction; then, the supernatant was further centrifuged at 1500× *g* for 40 min to the sediment and supernatant as the organelle fraction and cytosol fraction, respectively. The Sb content in three fractions was measured using an atomic fluorescence spectrometer (AFS-9760, Beijing, China).

### 2.5. ROS and RNS Detection

The distribution of H_2_O_2_, O_2_·^−^, NO, and ONOO^−^ in the roots were determined by the specific fluorescent probe H_2_DCFDA (2′,7′-dichlorodihydrofluorescein diacetate, Med Chem Express, Princeton, NJ, USA) [23], the superoxide anion fluorescent probe dihydroethidium (DHE, Biyuntian Biotechnology Co., Ltd., Shanghai, China) [24], the fluorescent probe DAF-FM DA (diaminofluorescein-FM diacetate, APExBIO, Houston, TX, USA) [25], and the fluorescent probe APF (3′-(p-aminophenyl) fluorescein, APExBIO, USA) [26], respectively. Images were captured using a fluorescence microscope (DYF-880, Dianying, Shanghai, China). H_2_O_2_ reacted with titanium sulfate and formed a peroxide–titanium complex precipitate; then, the precipitate was dissolved with concentrated sulfuric acid, and H_2_O_2_ content was estimated by measuring the characteristic absorption peak at 415 nm using a spectrophotometer (759 MC, Jinghua, Shanghai, China). The content of O_2_·^−^ (superoxide anion) was evaluated using the hydroxylamine oxidation method [27]. NO and ONOO^−^ images were processed and analyzed for quantification using ImageJ software (v2.1.4.7).

### 2.6. Detection of MDA Content and Cell Viability of Seedlings

The content of MDA was detected for the evaluation of membrane lipid peroxidation. MDA reacts with thiobarbituric acid (TBA) and forms a reddish-brown product under a high temperature and acidic conditions. A spectrophotometer was used to measure its characteristic absorption peak at 535 nm.

The viability of the root cells was monitored using the fluorescent dye fluorescein diacetate (FDA, Aladdin Reagent Co., Ltd., Shanghai, China) and propidium iodide (PI, Aladdin Reagent Co., Ltd., Shanghai, China) according to a method described in a previous study [28]. Images were captured using an inverted fluorescence microscope.

### 2.7. Antioxidant Enzyme Activities Determination

POD, SOD, CAT, and APX activities were determined using enzyme activity kits (Suzhou Grace Biotechnology Co., Ltd., Shuzhou, China). Briefly, fresh root samples were homogenized in chilled phosphate buffer (pH = 7.0) under liquid nitrogen and then centrifuged at 12,000 rpm at 4 °C for 15 min. The supernatant was collected, and the enzyme activities were measured according to instructions.

### 2.8. Estimation of Ascorbate–Glutathione Pool

AsA/DHA kits (Suzhou Grace Biotechnology Co., Ltd.) were used to determine root AsA and DHA contents. A total of 0.1 g fresh samples were grounded in liquid nitrogen and then homogenized with 1 mL of 5% (*w*/*v*) TCA (trichloroacetic acid) buffer before being centrifuged at 12,000 rpm for 10 min. The supernatant was then collected and measured for AsA and DHA content following the manufacturer’s instructions. GSH and GSSG contents were detected using GSH/GSSG kits (Beijing Solbio Technology Co., Ltd., Beijing, China). A total of 0.1 g fresh root samples were grounded and homogenized in sodium phosphate buffer (pH = 7.0) before being centrifuged at 8000× *g* and 4 °C for 10 min. The supernatant was collected to measure the GSH and GSSG contents according to the manufacturer’s instructions.

### 2.9. Statistical Analysis

SPSS 21.0 software (SPSS Inc., Chicago, IL, USA) was used to analyze all data. The mean values and standard deviations were calculated for each parameter measured in the study. A multiple comparisons test (Duncan’s SSP) was conducted for all different treatments (α = 0.05). The correlation analysis results between the indicators were analyzed for correlations (data from +Sb samples) using Origin 8.5 software. The Sb translocation factors (TFs) were estimated as follows according to our recent study [17]. The TFs of the Sb were estimated as follows: TFs _root−shoot_ = Sb content in shoots/Sb content in roots.

## 3. Results

### 3.1. Effects of MT on the Root Growth and Biomass of Rice, Sb Content, Transport Factors (TFs) of Sb, and Subcellular Distributions of Sb in Rice under Sb Stress

**Figure 1:**
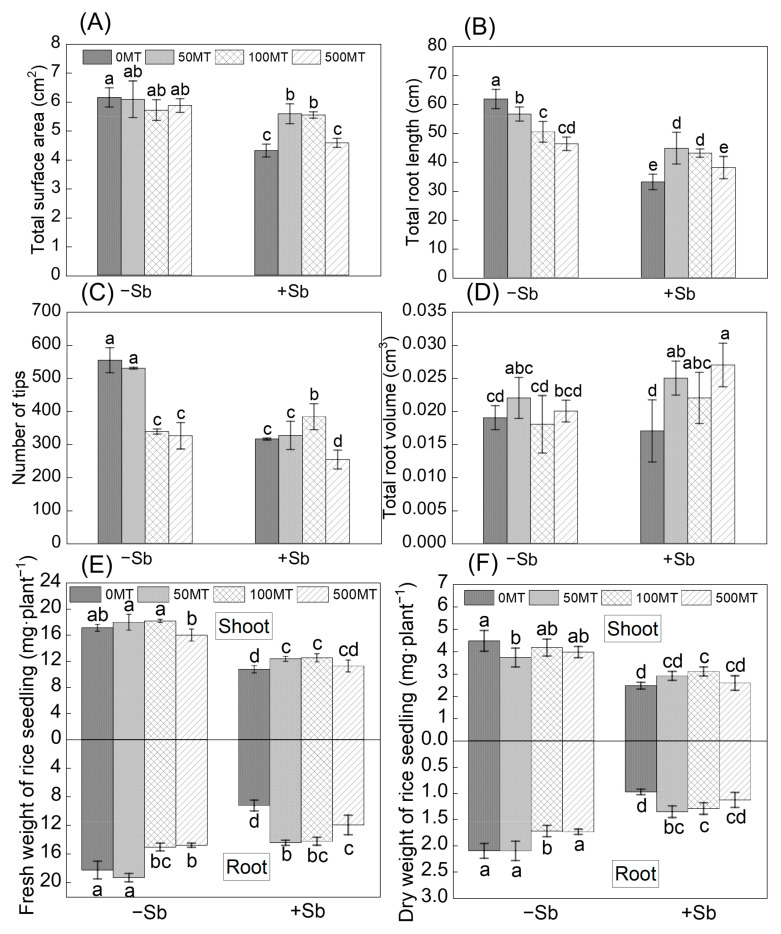
Effects of MT on the parameters of root growth: root total surface areas (**A**), root lengths (**B**), numbers of root tips (**C**), root volumes (**D**), fresh weights (**E**), and dry weights (**F**) of seedlings under Sb stress; 0 MT, 50 MT, 100 MT, 500 MT represent the concentration of MT application (0, 50, 100, and 500 µM, respectively); −Sb and +Sb mean no Sb or 50 µM Sb was added, respectively (the same as below). Multiple comparisons were conducted among different treatments (α = 0.05). The plotted columns are the mean values ± SD (*n* = 4). Different lowercase letters (a–e) indicate significant differences between two treatments.

MT treatments significantly decreased the root length and root tip numbers. The Sb alone treatment significantly decreased the root length, root total surface area, and root tip numbers by 46.5%, 29.9%, and 43.1%, respectively. Compared with the Sb alone treatment, the Sb + 50 MT and Sb + 100 MT treatments significantly increased the root length by 35.3% and 30.2% and total surface area by 29% and 28.6%, respectively. The Sb + 100 MT treatment significantly increased root tip numbers by 21.6% compared with the Sb alone treatment. The Sb + 50 MT, Sb + 100 MT, and Sb + 500 MT treatments significantly increased the root volumes by 35.3%, 30.7%, and 14.9% compared with the Sb alone treatment, respectively (Figure 1A–D). Biomass was measured based on the fresh and dry weight of the root samples (Figure 1E,F). The 100 MT and 500 MT treatments significantly decreased the fresh and dry weight of the roots. Sb stress significantly reduced the weight of both the shoots and roots. Compared with the Sb alone treatment, the Sb + 100 MT treatment significantly increased shoot and root dry weight by 25.7% and 32.7%, respectively.

**Figure 2:**
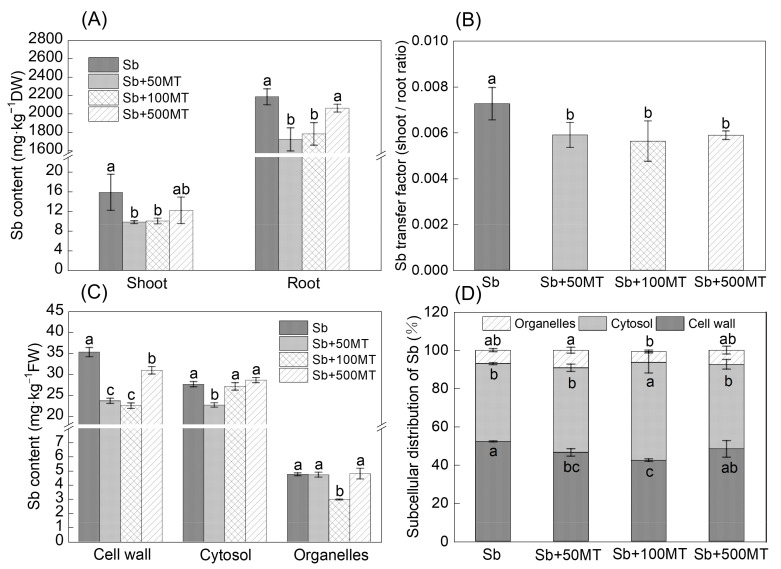
Effects of MT on total Sb content (**A**), TFs of Sb from roots to shoots (**B**), Sb content in subcellular fractions (**C**), and Sb subcellular distribution ratio (**D**) of rice seedlings under Sb stress. Sb, Sb + 50 MT, Sb + 100 MT, Sb + 500 MT means 50 µM Sb is added with 0, 50, 100, and 500 µM MT, respectively. For Figure 1A,C, the multiple comparisons were conducted among different treatments within the same part (α = 0.05); for Figure 1B,D, the multiple comparisons were conducted among the different treatments (α = 0.05). The plotted columns are mean values ± SD (*n* = 4). Different lowercase letters (a–c) indicate significant differences between two treatments.

Roots accumulated the most Sb; the Sb content in the roots was higher than that in the shoots. Sb + 50 MT and Sb + 100 MT significantly decreased shoot Sb content by 38.3% and 36.9%, respectively; the same treatments reduced root Sb content by 21.2% and 18.5%, respectively (Figure 2A). Sb + 50 MT, Sb + 100 MT, Sb + 500 MT significantly decreased the TFs of Sb from the roots to the shoots by 18.76%, 22.44%, and 18.93%, respectively (Figure 2B).

The Sb + 50 MT, Sb + 100 MT, and Sb + 500 MT treatments decreased Sb content in cell wall fractions by 33.01%, 36.3%, and 12.3%, respectively, compared with the Sb alone treatment (Figure 2C); Sb + 50 MT and Sb + 100 MT significantly decreased Sb content in cytosol and organelles by 18.1% and 37.7%, respectively. The subcellular distribution of Sb in most treatments was in the order of cell wall > cytosol > organelles, indicating that Sb was mainly accumulated in the cell walls and that the cell walls played a major role in tolerance to Sb stress. The proportion of Sb in the cell walls and cytoplasm varied in different treatments; the organelle fractions yielded the lowest proportion of Sb. The proportions of Sb in the cell walls, cytosol, and organelles were 52.2%, 40.8%, and 7.0%, respectively, in the Sb alone treatment (Figure 2B). Compared with the Sb alone treatment, the Sb + 50 MT and Sb + 100 MT treatments significantly decreased Sb proportion in the cell walls by 11.0% and 18.8%, respectively, while Sb + 100 MT significantly increased Sb proportion in the cell walls by 25.6% (Figure 2C,D). This suggests that MT significantly reduced the Sb content in the cell walls, while there were no effects on the Sb contents in cytosol and organelles.

### 3.2. Effects of MT on Oxidative Damage under Sb Stress in Seedlings

**Figure 3:**
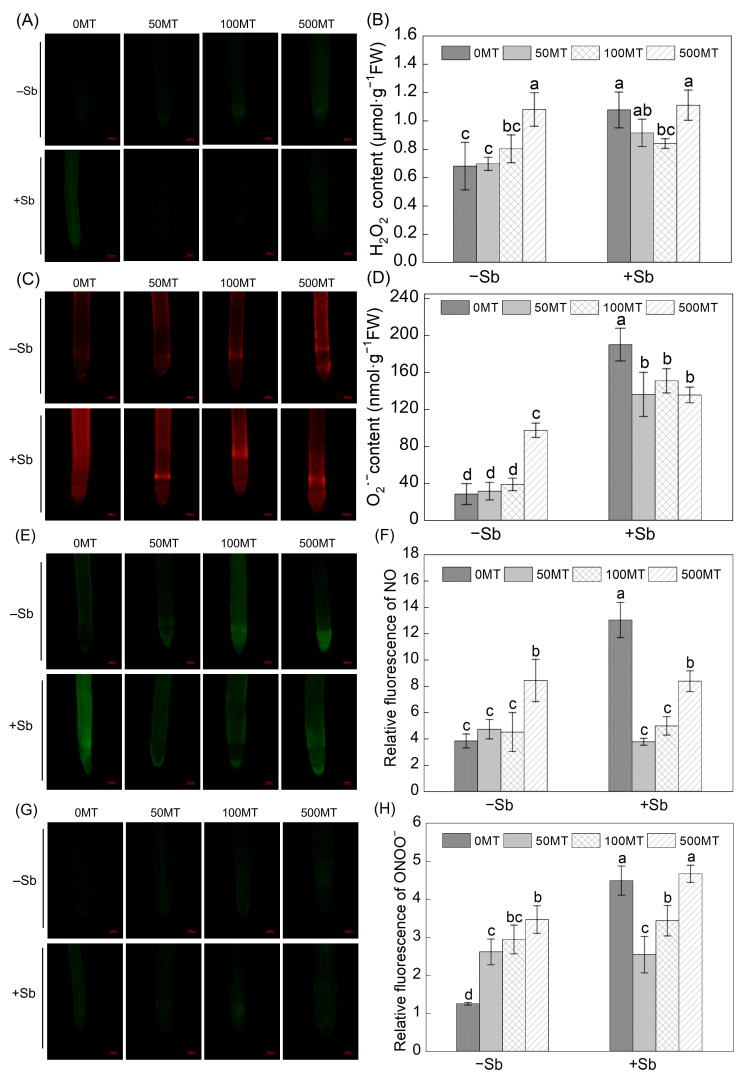
Effects of MT addition on ROS and RNS accumulation in seedling roots under Sb stress. Photographs of in vivo detection of H_2_O_2_ (**A**), O_2_·^−^ (**C**), NO (**E**), and ONOO^−^ (**G**) production were shown as red and green fluorescence in representative root tips, respectively; scale bar: 100 μM. O_2_·^−^ content (**B**), H_2_O_2_ content (**D**), and the average fluorescence intensity of NO (**F**) and ONOO^−^ (**H**) levels in the roots were quantified in arbitrary units. Multiple comparisons were conducted among the different treatments (α = 0.05). The plotted columns are mean values ± SD (*n* = 4). Different lowercase letters (a–d) indicate significant differences between two treatments.

**Figure 4:**
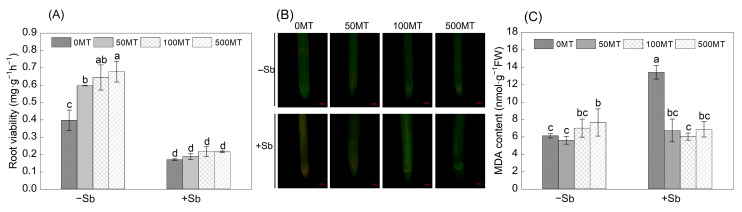
Effects of MT on root viability (**A**), cell viability (**B**), and MDA content (**C**) of roots under Sb stress. Scale bar: 100 μM. Multiple comparisons were conducted among different treatments (α = 0.05). The plotted columns are mean values ± SD (*n* = 4). Different lowercase letters (a–d) indicate significant differences between two treatments.

The ROS (Figure 3A–D), RNS (Figure 3E–H), root viability (Figure 4A), cell viability (Figure 4B), and root membrane lipid peroxidation (Figure 4C) of rice roots were detected to investigate whether MT has a mitigating effect on oxidative damage in root cells under Sb stress. The fluorescence staining results showed obvious solid green fluorescence and red fluorescence under the Sb alone treatment, the root tips also exhibited solid green fluorescence and red fluorescence (Figure 3A,C). The H_2_O_2_ and O_2_·^−^ contents (Figure 3B,D) were consistent with the results of the fluorescence staining. Sb stress increased H_2_O_2_ and O_2_·^−^ contents in the roots; the H_2_O_2_ and O_2_·^−^ contents were 0.8- and 4.8-fold that of the CK, respectively. The application of MT to the Sb-stressed seedlings greatly decreased the increases in H_2_O_2_ and O_2_^−^ in the roots compared with the Sb alone treatment; the Sb + 50 MT and Sb + 100 MT treatments reduced the H_2_O_2_ contents in the roots by 15.1% and 24.6%, respectively. Compared with the Sb alone treatment, the Sb + 50 MT, Sb + 100 MT and Sb + 500 MT treatments significantly reduced root O_2_·^−^ content by 28.4%, 20.6%, and 28.7%, respectively. This indicates that Sb stress increased the H_2_O_2_ and O_2_·^−^ contents in the roots, while exogenous MT application significantly reduced the contents of both H_2_O_2_ and O_2_·^−^ in the roots under Sb stress.

Regarding the image pixel intensity quantization for NO and ONOO^−^, compared with CK, the three MT treatments significantly elevated the accumulation of ONOO^−^ in the roots, but for ON accumulation, only the 500MT treatment showed a significant improving effect. The Sb alone treatment significantly increased NO and ONOO^−^ accumulation, and the NO and ONOO^−^ contents in the roots were 3.4 and 3.6 times higher than that of the control, respectively. The exogenous addition of 50 MT, 100 MT, and 500 MT significantly decreased the accumulation of NO in the roots under Sb stress. Except for the 500 MT treatment, the addition of 50 MT and 100 MT also caused a significant decrease in ONOO^−^ in the roots under Sb stress (Figure 3E,H).

Triphenyl tetrazolium chloride (TTC) is a molecule that detects dehydrogenase activity in living cells, and root vitality can be estimated from the reduction in TTC. Three MT treatments significantly increased the root viability by 50%, 41%, and 71%, respectively (Figure 4A). Sb stress significantly reduced root viability, and the Sb alone treatment decreased root viability by 57.3%. However, compared to the Sb alone treatment, the MT addition treatments did not exert any significant improvement effects on root viability. The heaviest red patches were observed in the Sb-treated rice roots, while MT attenuated the enhanced red fluorescence, which was induced by Sb stress (Figure 4B). The MDA content can reflect the degree of lipid oxidative damage in cell membranes. The 500 MT treatment significantly increased the MDA content in the roots. The root MDA content in the Sb alone treatment was 1.19 times that of CK. Compared with the Sb alone treatment, the Sb + 50 MT, Sb + 100 MT, and Sb + 500 MT treatments decreased root MDA content by 49.9%, 53.3%, and 48.9%, respectively (Figure 4C). These results indicate that MT could reduce the oxidative damage of rice under Sb stress.

### 3.3. Effects of MT on the Antioxidant Defense Systems of Seedlings under Sb Stress

**Figure 5:**
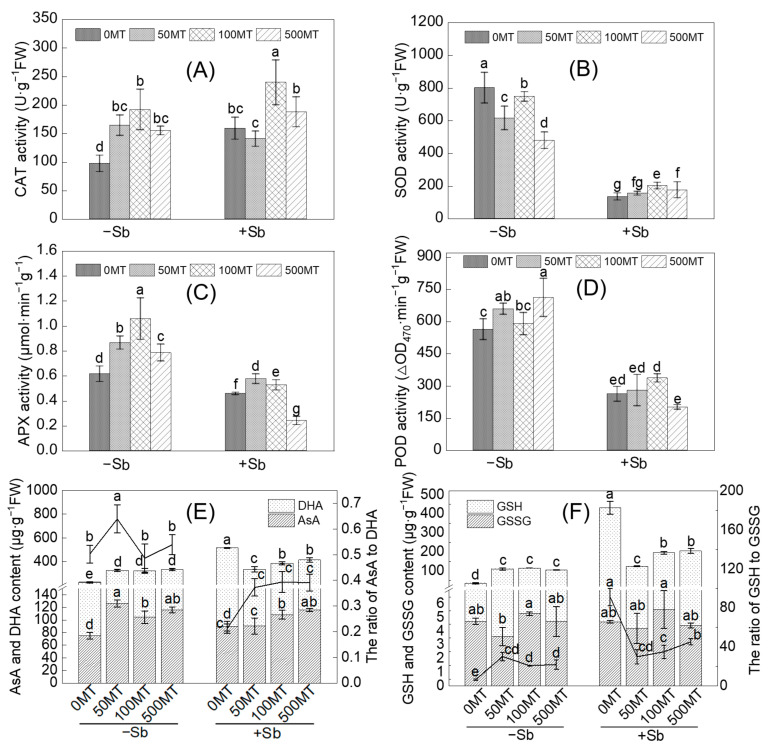
Effects of MT on CAT (**A**), SOD (**B**), APX (**C**), and POD (**D**) activities; AsA and GSH contents and AsA/DHA ratio (**E**); and GSH and GSSH contents and GSH/GSSG ratio (**F**) in the roots under Sb stress and the roots not under Sb stress. Multiple comparisons were conducted among the different treatments (α = 0.05). The plotted columns are mean values ± SD (*n* = 4). Different lowercase letters (a–g) indicate significant differences (*p* < 0.05) between two treatments.

The effect of MT on the Sb-induced oxidative detoxification of rice was studied by measuring the activity of antioxidant enzymes. The antioxidant enzymes activities (CAT, SOD, APX, and POD) in the roots showed that the effect of MT addition on antioxidant enzyme activities varied depending on the enzyme (Figure 5A–D). The MT treatments significantly increased root CAT, APX, and POD activities. All MT treatments significantly reduced SOD activity. The Sb alone treatment significantly reduced the activities of POD, SOD, and APX (by 25.5–53.4%) in the roots while significantly increasing CAT activity (by 63.5%). The Sb + 100 MT treatment significantly increased the activities of SOD, CAT, and APX in the roots by 50.3%, 50.7%, and 14.9% compared with the Sb alone treatment, respectively.

AsA and GSH are the most critical antioxidants in plant cells. The AsA, DHA, GSH, and GSSH contents in the roots were determined to investigated the change in the AsA–GSH cycle in response to the different treatments (Figure 5E). The AsA/DHA and GSH/GSSG ratios, which accurately represent the redox status of plant cells, were calculated to explore whether MT has a mitigating effect on oxidative damage from Sb stress in root cells (Figure 5F). All MT treatments significantly improved the root AsA and DHA contents. The AsA and DHA contents in the roots significantly increased by 20.1% and 184.1% in the Sb alone treatment, respectively, while all Sb treatments significantly decreased the AsA/DHA ratio. Compared with the Sb alone treatment, the Sb + 100 MT and Sb + 500 MT treatments significantly increased AsA content by 20.0% and 28.4%, respectively, while the Sb + 50 MT, Sb + 100 MT and Sb + 500 MT treatments significantly reduced DHA content by 42.9%, 35.1%, and 30.0%, respectively. MT addition also significantly increased the ratio of AsA to DHA under Sb stress.

The MT treatments with no Sb addition significantly increased GSH content and GSH/GSSH ratio in the roots. The Sb + 50 MT, Sb + 100 MT, and Sb + 500 MT treatments significantly reduced the root GSH content (by 53.1–71.8%) and GSH/GSSG ratio compared with the Sb alone treatment. These results indicated that MT was beneficial in activating the antioxidant defense system, maintaining redox balance, and improving the antioxidant capacity of the rice under Sb stress conditions.

### 3.4. Correlation Analysis between Enzyme and Non-Enzyme Indexes of Antioxidant System, Active Oxygen Contents, Sb Content, Subcellular Distribution of Sb, Root Vitality, and Biomass of Rice

**Figure 6:**
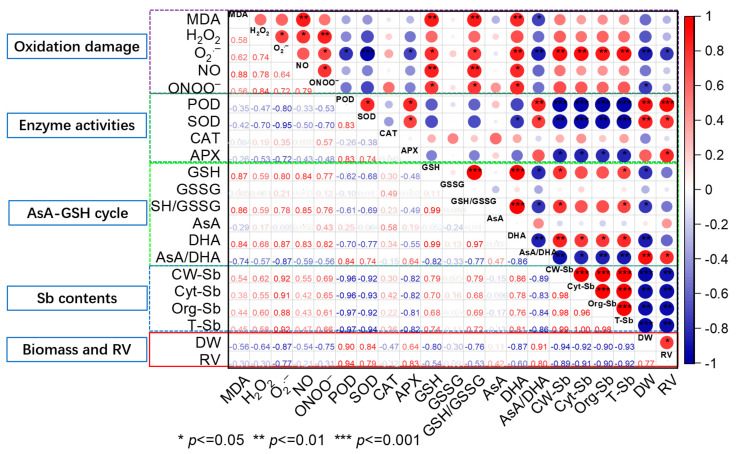
Correlation matrix between enzyme and non-enzyme indexes of antioxidant system, active oxygen contents, Sb content, subcellular distribution of Sb, root vitality, and biomass of seedlings. Notes: RV, CW-Sb, Cyt-Sb, Org-Sb, and T-Sb represent root validity, Sb content in cell walls, Sb content in cytosol, Sb content in organelles, and total Sb content in roots, respectively; the blue and red circles indicate a negative or positive correlation between two indexes, respectively; “***”, “**”, and “*” indicate *p* ≤ 0.001, *p* ≤ 0.01, and *p* ≤ 0.05, respectively.

The variations in biomass (dry weight) and root vitality were positively correlated with the variations in AsA/DHA ratio and APX, SOD, and POD activities, explaining nearly over 80% of the variation, while dry weight of seedling and root vitality were negatively correlated with the variations in Sb content (subcellular fractions Sb and total Sb) and O_2_·^−^ content, explaining 77–94% of the variation. In addition, the biomass of rice was also negatively correlated with the DHA and GSH contents, GSH/GSSH ratio, and ONOO^−^ content, which explained 76–87% of the observed variation. Multiple antioxidant enzyme activities of defense systems (POD, SOD, and APX) were negatively correlated with O_2_·^−^ content, explaining 72–95% of the variation. However, as the major non-enzymatic antioxidants, the contents of GSH and DHA and GSH/GSSH ratio were positively correlated with the many indexes of oxidative damage, including the MDA, O_2_·^−^, NO, and ONOO^−^ contents, which explained 76–87% of the observed variation. The subcellular Sb fractions and total Sb were positively correlated with the O_2_·^−^ and DHA contents, explaining 76–92% of the variation, while they were negatively correlated with the POD, SOD, and APX activities and AsA/HA ratio, explaining 81–97% of the variation. Additionally, the subcellular fractions of Sb and total Sb were positively correlated with GSH content and GSH/GSSH ratio, explaining 72–79% of the variation (Figure 6).

## 4. Discussion

### 4.1. MT Alleviates the Inhibition of Sb Stress on the Growth of Rice

The excessive accumulation of Sb can harm plants by inhibiting their growth, preventing the absorption of mineral elements [29]. Plants can respond to heavy metal stress by altering root growth patterns and morphology [6]. Sb(III) exposure led to a significant reduction in root growth parameters in a previous study [7]. In this study, MT application mitigated the inhibition of Sb on rice growth; similar results were also found in naked oat and rice seedlings subjected to Cd and As stress, respectively [30,31]. The Sb treatments significantly decreased the biomass, root growth parameters (including the length and surface area of the roots and root tip numbers) (Figure 1), and root viability, consistent with the findings of Luo et al. [32]. The Sb + MT treatments significantly increased root growth patterns by 20–35%, and the Sb + 100 MT treatment significantly increased the shoot or root weight by 16.5–53.8% (Figure 1). Root viability responds to the oxidative, reductive, and synthetic capacities of plant roots and indirectly measures the growth of roots and metabolic functions [33]. MT application in rice under Sb stress (Sb + 50 MT and Sb + 100 MT) significantly improved root viability by nearly 30% (Figure 4A), as measured by the percentage of live roots. This shows that exogenous MT was beneficial in promoting rice growth under Sb stress.

### 4.2. MT Reduces Sb Accumulation in Rice Seedlings

Sb is similar to As, which is easily absorbed by plants, and most of it is accumulated in rice roots. Exogenous MT significantly decreased the Sb content in rice under Sb stress and significantly decreased the TFs of Sb (Figure 2). This indicates that MT application reduced the absorption and transportation of Sb in the rice under Sb stress, thus alleviating Sb toxicity, and these results are similar to the results of an earlier study [34]. In addition, the cell walls and cytosol fractions of the roots are the main enrichment sites of Sb (Figure 2). Another study also showed that the highest and the lowest Sb content was found separately in the root cell walls and organelles [9]. Cell walls can store or isolate heavy metals; they are the first barrier heave metals face when entering a root cell, and they play an essential role in reducing the toxicity of heavy metals to plants [35]. The pectin in the root cell walls inhibit extracellular Sb [7]. The application of 50 and 100 µM MT under Sb stress significantly decreased the proportion of Sb in the cell walls, while the Sb + 100 MT treatment significantly increased the proportion of Sb in cytosol (Figure 2C,D). It has been suggested that As could be fixed with sulfhydryl-containing active substances such as GSH and PCs [36] and ultimately be isolated in the vacuoles of plant cells. Sb is similar to As in terms of its chemical properties; Sb may be fixed with GSH or PCs to form Sb-PCs or Sb-GS complexes, thus decreased the proportion of Sb in the cell walls. Additionally, MT might alter the structure and composition of the cell walls, affect the ability of the cell walls to store the amount of Sb, and, consequently, reduce the proportion of Sb in the cell walls, as it has been pointed that MT contributes to the elimination of Al from wheat root tips by reducing the content of polysaccharides and pectin demethylation in the roots, thus reducing Al accumulation in wheat roots [27].

### 4.3. MT Alleviates Oxidative Damage in Seedling Roots under Sb Stress

In this study, Sb stress led to a significant accumulation of ROS, RNS, and MDA in rice root cells. The H_2_O_2_, O_2_·^−^, NO, ONOO^−^, and MDA contents in roots were 0.8-, 4.8-, 3.4-, 3.6-, and 1.19-fold that of the control, respectively (Figure 3 and Figure 4C). The subcellular fractions of Sb and total Sb were significantly correlated with ROS (O_2_·^−^) (Figure 6). As basic signals of plants, ROS and RNS are involved in regulating plant metabolism, growth, and development, as well as the response to abiotic stresses [37]. Under normal conditions, excess ROS in rice cells can be removed via its antioxidant defense mechanism. However, Sb stress disturbs the balance between ROS production and removal. Rice seedling cells show a dramatic increase in ROS and disrupt cell structure, which will be associated with an increase in RNS acting on the antioxidant system [11]. Sb stress also causes oxidative damage in other plants. For example, the excessive accumulation of Sb leads to growth impairment and increased ROS and RNS in tomatoes [10] and also induces membrane lipid peroxidation in wheat plants [38]. It has been found that Cd induced ONOO^−^ accumulation in soybean seedlings, which was accompanied by increased NO and O_2_·^−^ levels [39]. MT application can directly mitigate the toxicity caused by heavy metals by scavenging ROS and RNS [13]. MT application significantly reduced the content of ROS, RNS, and MDA in the roots in this study, as the addition of 100 µM MT under Sb stress reduced H_2_O_2_ by 24.6%; MT addition under Sb stress significantly decreased the O_2_·^−^ contents by nearly 20–30%, caused a significant reduction in the contents of NO and ONOO^−^ (Figure 3), and nearly decreased MDA content by 50% (Figure 4C). These results show that the addition of exogenous MT could reduce the oxidative damage and cell membrane damage caused by Sb stress in rice.

Plants scavenge accumulated ROS and RNS effectively through the synergistic action of antioxidant enzymes such as CAT, SOD, POD, and APX. In the present study, Sb stress decreased the POD, SOD, and APX activities of the roots (by 25–80%). However, MT application under Sb stress (Sb + 100 MT) increased the SOD, POD, CAT, and APX activities in the roots by 15–50% (Figure 5). The subcellular fractions Sb and total Sb were negatively correlated with the activities of POD, SOD, and APX (Figure 6). These results suggest that MT is involved in reducing oxidative damage to the cell membrane by improving the activities of antioxidant enzymes. Another study also found that Sb stress increased SOD activity and reduced POD activity, with no significant effect on APX activity in rice plants [7]. The addition of exogenous MT has been shown to improve the activities of the key antioxidant enzymes of tea plants under As stress [40]. In addition, MT addition has been shown to improve antioxidant properties and Cd-induced oxidative stress in mushrooms through glutathione metabolism, redox processes, and cellular oxidative detoxification [41]. This indicates that MT reduces ROS levels in rice roots by stimulating the antioxidant system and increasing antioxidant enzyme activity and antioxidant potential under Sb stress, thus improving plant tolerance to Sb stress [40].

Sb treatment significantly increased the contents of AsA and DHA, the GSH content, and the ratio of GSH to GSSH while significantly decreasing the ratio of AsA to DHA in the roots (Figure 5). The variation in these indicators possibly suggests that Sb stress disrupted the original redox balance of the AsA-GSH cycle in the roots system, which was similar to the effects of Sb on the AsA and GSH levels in sunflower roots [42]. GSH is the most important metabolite in the antioxidant defense system of plants [43], and the detoxification of thiol groups within GSH forming complexes with Sb may be one of the reasons for the imbalance of antioxidant components. MT application under Sb stress significantly increased AsA content (by 20.0–28.4%) and the ratio of AsA to DHA while significantly reducing DHA and GSH contents (over 50%) and the ratio of GSH to GSSH in the roots (Figure 5), and a similar result was also reported in recent studies [20,44], in which MT increased AsA content and decreased GSH content in safflower seedlings under Zn stress and in wheat under Cd stress. The Sb content in cell wall and total Sb was positively correlated with DHA and GSH content and GSH/GSSH ratio, while they were negatively correlated with AsA/DHA ratio (Figure 6). This potentially indicates that MT was beneficial in activating the antioxidant defense system, maintaining the dynamic of the AsA-GSH cycle redox balance, and improving the antioxidant capacity of rice under Sb stress. AsA and GSH, as the predominant non-enzymatic antioxidants, can improve the oxidative capacity of plants to reduce oxidative damage by abiotic stress. GSH positively regulates several transcription factors associated with stress response genes and antioxidant enzyme activities [45]. The AsA-GSH cycle plays a key role in removing and controlling ROS or RNS produced under stress conditions, balancing ROS or RNS synthesis and scavenging, and preventing cellular damage, as it has been suggested that AsA scavenges ONOO^−^, and high concentrations of AsA and GSH maintain low levels of derivatives of NO in plants [46]. MT may stimulate the production of phytochelatins (PCs) under Sb stress to alter cellular redox homeostasis [47], increasing the depletion of GSH content and thus decreasing the GSH content in the Sb + MT treatments compared with the Sb alone treatment. In addition, key enzymes in the AsA-GSH cycle are also susceptible to changing their activities due to oxidative damage, thus disrupting oxidative defense, which is related to plant species and developmental stage. Different defense mechanisms may be determined by different Sb stress concentrations [45]; however, the specific mechanism underlying this process requires further study.

### 4.4. Pathways of Each Factor on Seedling Biomass Based on Structural Equation Modeling (SEM)

**Figure 7:**
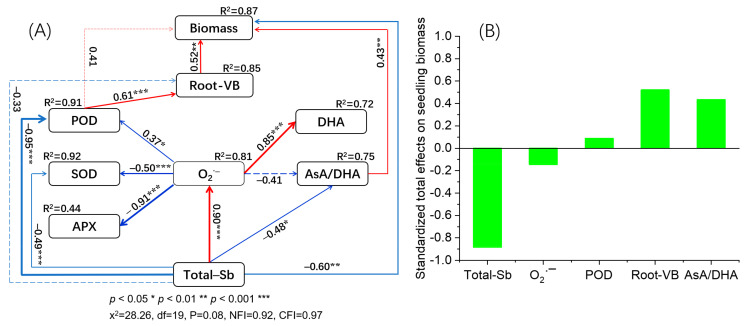
The direct and indirect effects of root viability; POD, SOD, and APX actives; O_2_·^−^, DHA content; AsA/DHA ratio; and total Sb content on biomass based on structural equation modeling (**A**) and the standardized total effects (direct + indirect) on biomass (**B**). Positive and negative correlations are indicated by red and blue arrows, respectively. Real and virtual arrows represent significant and insignificant relationships, respectively. The number next to the arrow is the path coefficient; the thickness of the arrow indicates the size of the path coefficient. “***”, “**”, and “*” indicate *p* ≤ 0.001, *p* ≤ 0.01, and *p* ≤ 0.05, respectively.

SEM showed good fit to the biomass, as indicated by the x^2^, df, and GFI values (Figure 7A). The model explained 87% of the variance in the biomass values. Root viability (path coefficient (pc): 0.52, *p* < 0.01), total Sb content (pc: 0.60, *p* < 0.01), and the ratio of AsA to DHA (pc: 0.43, *p* < 0.01) had direct associations with the biomass values. The direct effects on biomass were root viability (pc: 0.52, *p* < 0.01), total Sb content (pc: 0.60, *p* < 0.01), and the ratio of AsA to DHA (pc: 0.43, *p* < 0.01). POD indirectly affected biomass via affecting root viability (pc: 0.52–0.61, *p* < 0.01). O_2_·^−^ influenced biomass indirectly via affecting POD and root viability (pc: 0.37–0.61, *p* < 0.05). In addition, total Sb content affected biomass indirectly via O_2_·^−^, POD, root viability, and AsA/DHA ratio (pc: 0.34–0.93, *p* < 0.05). The standardized total effects (Figure 7B) showed that biomass was mainly affected by Sb content, followed successively by root viability, AsA/DHA ratio, O_2_·^−^, and POD active. This indicates that O_2_·^−^ content is the most important response index, as it reflected the degree of oxidation damage caused by Sb stress in this study. MT alleviated oxidation damage mainly through regulating POD activity and AsA/DHA ratio; root viability was subsequently affected, and MT also regulated the rice growth. Our data clearly show that MT application alleviated the oxidative damage of rice seedlings under Sb stress by regulating key antioxidant enzymes maintain in proper levels in the AsA-DHA cycle.

## 5. Conclusions

The application of MT reduced the entry of Sb into the cell walls and decreased the accumulation of Sb in the rice roots, thus significantly improving the growth parameters and vitality of the roots. MT enhanced the antioxidant defense system through up-regulating the activity of antioxidant enzymes to reduce the production of reactive oxygen species and subsequent lipid peroxidation to enhance tolerance to Sb stress. In addition, MT improved the efficiency of the AsA-GSH cycle, thereby re-establishing the cellular redox state. The results of this study reveal the critical mechanisms by which MT reduces Sb accumulation and alleviates Sb toxicity in rice, indicating that exogenous MT should be a potential effective way to improve the resistance of rice to Sb stress. Further studies should consider whether the crosstalk between MT and other hormones exerts an effect on relieving Sb stress.

## Data Availability

Data are contained within the article and Supplementary Materials.

## Supplementary Materials

The following supporting information can be downloaded at: https://www.mdpi.com/article/10.3390/antiox12111917/s1, Supplementary Table S1: Effects of different concentrations of Sb on the length, fresh weight, and dry weight of the rice.

## References

[1] Long X.J., Wang X., Guo X.J., He M.C. (2020). A review of removal technology for antimony in aqueous solution. J. Environ. Sci..

[2] Liu Y.Q., Lv W.X., Zhao Z.Q., Yang Y.P., Zhang L.X., Wang L.Y., Jiang C.Y., Duan G.L., Zhu Y.G. (2022). Aluminum adsorption and antimonite oxidation dominantly regulate antimony solubility in soils. Chemosphere.

[3] He M., Wang N., Long X., Zhang C., Ma C., Zhong Q., Wang A., Wang Y., Pervaiz A., Shan J. (2019). Antimony speciation in the environment: Recent advances in understanding the biogeochemical processes and ecological effects. J. Environ. Sci..

[4] Andreo-Jimenez B., te Beest D.E., Kruijer W., Vannier N., Kadam N.N., Melandri G., Jagadish S.V.K., van der Linden G., Ruyter-Spira C., Vandenkoornhuyse P. (2023). Genetic mapping of the root mycobiota in rice and its role in drought tolerance. Rice.

[5] Feng R.W., Liao G.J., Guo J.K., Wang R.G., Xu Y.M., Ding Y.Z., Mo L.Y., Fan Z.L., Li N. (2016). Responses of root growth and antioxidative systems of paddy rice exposed to antimony and selenium. Environ. Exp. Bot..

[6] Zhu Y.M., Wu Q.H., Lv H.Q., Chen W.X., Wang L.Z., Shi S.J., Yang J.G., Zhao P.P., Li Y.P., Christopher R. (2020). Toxicity of different forms of antimony to rice plants: Effects on reactive oxidative species production, antioxidative systems, and uptake of essential elements. Environ. Pollut..

[7] Feng R.W., Lei L., Su J.M., Zhang R.R., Zhu Y.M., Chen W.X., Wang L.Z., Wang R.J., Dai J.X., Lin Z.T. (2020). Toxicity of different forms of antimony to rice plant: Effects on root exudates, cell wall components, endogenous hormones and antioxidant system. Sci. Total Environ..

[8] Vidya C.S.N., Shetty R., Vaculíková M., Vaculík M. (2022). Antimony toxicity in soils and plants, and mechanisms of its alleviation. Environ. Exp. Bot..

[9] Zhu Y., Li Z., Shen J., Wu K., Zhao P., Wu Z., Liu Z., Yang J., Liu H., Rensing C. (2022). Toxicity of different forms of antimony to rice plants: Photosynthetic electron transfer, gas exchange, photosynthetic efficiency, and carbon assimilation combined with metabolome analysis. J. Hazard. Mater..

[10] Espinosa-Vellarino F.L., Garrido I., Ortega A., Casimiro I., Espinosa F. (2020). Effects of antimony on reactive oxygen and nitrogen species (ROS and RNS) and antioxidant mechanisms in tomato plants. Front. Plant Sci..

[11] Espinosa-Vellarino F.L., Garrido I., Ortega A., Casimiro I., Espinosa F. (2021). Response to antimony toxicity in dittrichia viscosa plants: ROS, NO, H_2_S, and the antioxidant system. Antioxidants.

[12] Anjum N.A., Ahmad I., Mohmood I., Pacheco M., Duarte A.C., Pereira E., Umar S., Ahmad A., Khan N.A., Iqbal M. (2011). Modulation of glutathione and its related enzymes in plants’ responses to toxic metals and metalloids—A review. Environ. Exp. Bot..

[13] Pardo-Hernandez M., Lopez-Delacalle M., Marti-Guillen J.M., Martinez-Lorente S.E., Rivero R.M. (2021). ROS and NO phytomelatonin-induced signaling mechanisms under metal toxicity in plants: A review. Antioxidants.

[14] Hoque M.N., Tahjib-Ul-Arif M., Hannan A., Sultana N., Akhter S., Hasanuzzaman M., Akter F., Hossain M., Sayed M.A., Hasan M.T. (2021). Melatonin modulates plant tolerance to heavy metal stress: Morphological responses to molecular mechanisms. Int. J. Mol. Sci..

[15] Ni J., Wang Q.J., Shah F.A., Liu W.B., Wang D.D., Huang S.W., Fu S.L., Wu L.F. (2018). Exogenous melatonin confers cadmium tolerance by counterbalancing the hydrogen peroxide homeostasis in wheat seedlings. Molecules.

[16] Jiang Y., Huang S.H., Ma L., Kong L.L., Pan S.G., Tang X.R., Tian H., Duan M.Y., Mo Z.W. (2022). Effect of exogenous melatonin application on the grain yield and antioxidant capacity in aromatic rice under combined lead-cadmium stress. Antioxidants.

[17] Bao Q.L., Bao W.K., Li Y., Zhang S.N., Lian F., Huang Y.Z. (2021). Silicon combined with foliar melatonin for reducing the absorption and translocation of Cd and As by *Oryza sativa* L. in two contaminated soils. J. Environ. Manag..

[18] Lei K.Q., Sun S.Z., Zhong K.T., Li S.Y., Hu H., Sun C.J., Zheng Q.M., Tian Z.W., Dai T.B., Sun J.Y. (2021). Seed soaking with melatonin promotes seed germination under chromium stress via enhancing reserve mobilization and antioxidant metabolism in wheat. Ecotoxicol. Environ. Saf..

[19] Jahan M.S., Guo S.R., Baloch A.R., Sun J., Shu S., Wang Y., Ahammend G.J., Kabir K., Roy R. (2020). Melatonin alleviates nickel phytotoxicity by improving photosynthesis, secondary metabolism and oxidative stress tolerance in tomato seedlings. Ecotoxicol. Environ. Saf..

[20] Goodarz A., Namdjoyan S., Soorki A.A. (2020). Effects of exogenous melatonin and glutathione on zinc toxicity in safflower (*Carthamus tinctorius* L.) seedlings. Ecotoxicol. Environ. Saf..

[21] Li L., Yan Y.X. (2021). Insights into the roles of melatonin in alleviating heavy metal toxicity in crop. Phyton-Int. J. Exp. Bot..

[22] Wang Y., Chai L.Y., Yang Z.H., Mubarak H., Xiao R.Y., Tang C.J. (2017). Subcellular distribution and chemical forms of antimony in *Ficus tikoua*. Int. J. Phytoremediation.

[23] Chen Z.P., Gu Q., Yu X.L., Huang L.Q., Xu S., Wang R., Shen W., Shen W.B. (2018). Hydrogen peroxide acts downstream of melatonin to induce lateral root formation. Ann. Bot..

[24] Adhikari S., Ghosh S., Azahar I., Adhikari A., Shaw A.K., Konar S., Roy S., Hossain Z. (2018). Sulfate improves cadmium tolerance by limiting cadmium accumulation, modulation of sulfur metabolism and antioxidant defense system in maize. Environ. Exp. Bot..

[25] Singh S., Prasad S.M. (2019). Management of chromium (VI) toxicity by calcium and sulfur in tomato and brinjal: Implication of nitric oxide. J. Hazard. Mater..

[26] Luo B.F., Du S.T., Lu K.X., Liu W.J., Lin X.Y., Jin C.W. (2012). Iron uptake system mediates nitrate-facilitated cadmium accumulation in tomato (*Solanum lycopersicum*) plants. J. Exp. Bot..

[27] Sun C.L., Lv T., Huang L., Liu X.X., Jin C.W., Lin X.Y. (2020). Melatonin ameliorates aluminum toxicity through enhancing aluminum exclusion and reestablishing redox homeostasis in roots of wheat. J. Pineal Res..

[28] He J.M., Ma X.G., Zhang Y., Sun T.F., Xu F.F., Chen Y.P., Liu X., Yue M. (2013). Role and interrelationship of Gα protein, hydrogen peroxide, and nitric oxide in ultraviolet B-induced stomatal closure in arabidopsis leaves. Plant Physiol..

[29] Altaf M.A., Shahid R., Ren M., Altaf M.M., Jahan M.S., Khan L.U. (2021). Melatonin mitigates nickel toxicity by improving nutrient uptake fluxes, root architecture system, photosynthesis, and antioxidant potential in tomato seedling. J. Soil Sci. Plant Nutr..

[30] Wang K., He J.J., Gao Y., Han K., Liu J.Q., Wang Y.J. (2022). Exogenous melatonin improved the growth and development of naked oat seedlings under cadmium stress. Environ. Sci. Pollut. Res. Int..

[31] Zhang S.N., Bao Q.L., Huang Y.Z., Han N. (2022). Exogenous plant hormones alleviate As stress by regulating antioxidant defense system in *Oryza sativa* L. Environ Sci Pollut Res Int. Environ. Sci. Pollut. Res..

[32] Luo W.T., He L., Li F., Li J.K. (2020). Exogenous salicylic acid alleviates the antimony (Sb) toxicity in rice (*Oryza sativa* L.) seedlings. J. Plant Growth Regul..

[33] Dong Y.M., Gao M.L., Song Z.G., Qiu W.W. (2020). Microplastic particles increase arsenic toxicity to rice seedlings. Environ. Pollut..

[34] Samanta S., Banerjee A., Roychoudhury A. (2021). Exogenous melatonin regulates endogenous phytohormone homeostasis and thiol-mediated detoxification in two indica rice cultivars under arsenic stress. Plant Cell Rep..

[35] Berni R., Luyckx M., Xu X., Legay S., Sergeant K., Hausman J.F., Lutts S., Cai G., Guerriero G. (2019). Reactive oxygen species and heavy metal stress in plants: Impact on the cell wall and secondary metabolism. Environ. Exp. Bot..

[36] Tuli R., Chakrabarty D., Trivedi P.K., Tripathi R.D. (2010). Recent advances in arsenic accumulation and metabolism in rice. Mol. Breed..

[37] Turkan I. (2018). ROS and RNS: Key signalling molecules in plants. J. Exp. Bot..

[38] Ma C., He M., Zhong Q., Ouyang W., Lin C., Liu X. (2019). Uptake, translocation and phytotoxicity of antimonite in wheat (*Triticum aestivum*). Sci. Total Environ..

[39] Gzyl J., Izbiańska K., Floryszak-Wieczorek J., Jelonek T., Arasimowicz-Jelonek M. (2016). Cadmium affects peroxynitrite generation and tyrosine nitration in seedling roots of soybean (*Glycine max* L.). Environ. Exp. Bot..

[40] Li X., Ahammed G.J., Zhang X.N., Zhang L., Yan P., Zhang L.P., Fu J.Y., Han W.Y. (2021). Melatonin-mediated regulation of anthocyanin biosynthesis and antioxidant defense confer tolerance to arsenic stress in *Camellia sinensis* L.. J. Hazard. Mater..

[41] Gao Y.Y., Wang Y., Qian J., Si W.S., Tan Q., Xu J.Y., Zhao Y.C. (2020). Melatonin enhances the cadmium tolerance of mushrooms through antioxidant-related metabolites and enzymes. Food Chem..

[42] Ortega A., Garrido I., Casimiro I., Espinosa F. (2017). Effects of antimony on redox activities and antioxidant defence systems in sunflower (*Helianthus annuus* L.) plants. PLoS ONE.

[43] Koh Y.S., Wong S.K., Ismail N.H., Zengin G., Duangjai A., Saokaew S., Phisalprapa P., Tan K.W., Goh B.H., Tang S.Y. (2021). Mitigation of environmental stress-impacts in plants: Role of sole and combinatory exogenous application of glutathione. Front. Plant Sci..

[44] Li G.Z., Wang Y.Y., Liu J., Liu H.T., Liu H.P., Kang G.Z. (2022). Exogenous melatonin mitigates cadmium toxicity through ascorbic acid and glutathione pathway in wheat. Ecotoxicol. Environ. Saf..

[45] Hasanuzzaman M., Bhuyan M., Anee T.I., Parvin K., Nahar K., Mahmud J.A., Fujita M. (2019). Regulation of ascorbate-glutathione pathway in mitigating oxidative damage in plants under abiotic stress. Antioxidants.

[46] Meng X., Luo S., Dawuda M.M., Gao X., Wang S., Xie J., Tang Z., Liu Z., Wu Y., Jin L. (2021). Exogenous silicon enhances the systemic defense of cucumber leaves and roots against CA-induced autotoxicity stress by regulating the ascorbate-glutathione cycle and photosystem II. Ecotoxicol. Environ. Saf..

[47] Riyazuddin R., Nisha N., Ejaz B., Khan M.I.R., Kumar M., Ramteke P.W., Gupta R. (2021). A comprehensive review on the heavy metal toxicity and sequestration in plants. Biomolecules.

