# Supplementary material for: Melatonin Alleviates Antimony Toxicity by Regulating the Antioxidant Response and Reducing Antimony Accumulation in Oryza sativa L
Source: Antioxidants (Basel). 2023 Oct 26;12(11):1917. doi: 10.3390/antiox12111917 (PMC10669696; doi:10.3390/antiox12111917)
Supplement: Supplementary file 1 [file antioxidants-12-01917-s001.zip › antioxidants-2662459-supplementary.pdf]

Supporting Information

Melatonin alleviates antimony toxicity by reducing antimony accumulation and regulating the antioxidant response in *Oryza sativa* L.

Materials and Methods

Plant materials and treatment

Select plump rice seeds, disinfect them with a 5% hydrogen peroxide (H_2O_2) solution for 15 min, and then rinse them with deionized water. The disinfected and washed seeds were evenly sown in seedling trays and placed in a constant temperature incubator at 28°C for germination. Select seeds with a consistent white appearance for transplanting into black seedling boxes (10.5 cm × 15.5 cm × 17.0 cm, L × W × H) and grow them in an artificial climate chamber. Rice seedlings were treated when the roots were about 1 cm long. The total six Sb concentrations of 0, 10, 30, 50, 100, or 150 μM were set for Sb stress. Sb was added as $C_8H_4K_2O_{12}Sb_2{\cdot}3H_2O$ to the corresponding nutrient solution. Each treatment contained three biological replicates arranged in randomized blocks. During the treating period, the culture solution was changed every two days. After one week of treatment, rice seedlings were harvested and rinsed with deionized water for the next analysis. Seedlings were divided into root and aboveground parts, measured with a ruler to determine their length, and weighed with an analytical balance to determine their fresh weight (FW) and dry weight (DW). Seedlings were dried in an oven maintained at 80 °C for 48 h before recording dry weight.

The results showed that the 50 μM Sb significantly affected the biomass of rice seedlings compared with CK, thus it was selected as the Sb stress concentration in the formal experiment.

Table S1. Effects of different concentrations of Sb on the length, fresh weigh and dry weight of the rice seedlings

Treatments	Shoot			Root		
	Shoot height (cm)	Fresh weight (mg plant^{-1})	Dry weight (mg plant^{-1})	Root height (cm)	Fresh weight (mg plant^{-1})	Dry weight (mg plant^{-1})

CK	5.79a±0.41	19.51a±1.24	3.74a±0.24	15.38a±1.14a	18.32b±1.23	2.11ab±0.14
10μM Sb	4.51b±0.39	14.61b±0.44	3.78a±0.23	12.2b±0.88	20.72a±1.75	2.54a±0.22
30μM Sb	4.04c±0.35	15.81b±0.45	3.1b±0.20	10.49c±0.5	18.73b±0.34	2.28ab±0.03
50μM Sb	3.53d±0.31	14.65b±0.8	3.07b±0.30	8.84d±0.57	9.30c±0.75	1.92b±0.49
100μM Sb	3.02e±0.17	11.48c±1.19	2.39c±0.29	4.81e±0.41	9.12c±0.97	1.09c±0.17
150μM Sb	2.83e±0.31	9.81d±0.86	2.33c±0.19	3.73f±0.63	9.0c2±0.5	1.12c±0.03

Notes: The multiple comparisons were conducted among different treatments (α = 0.05). The values are mean ± SD (n = 4). Different low-case letters (a, b, c, d, e, and f) show the significant differences between two treatments.
